# Supplementary material for: Analysis of the Composition of Deinagkistrodon acutus Snake Venom Based on Proteomics, and Its Antithrombotic Activity and Toxicity Studies
Source: Molecules. 2022 Mar 29;27(7):2229. doi: 10.3390/molecules27072229 (PMC9000436; doi:10.3390/molecules27072229)
Supplement: Supplementary file 1 [file molecules-27-02229-s001.zip › molecules-1609315-supplementary/Supplemental Figure S1.pptx]

## Slide 1
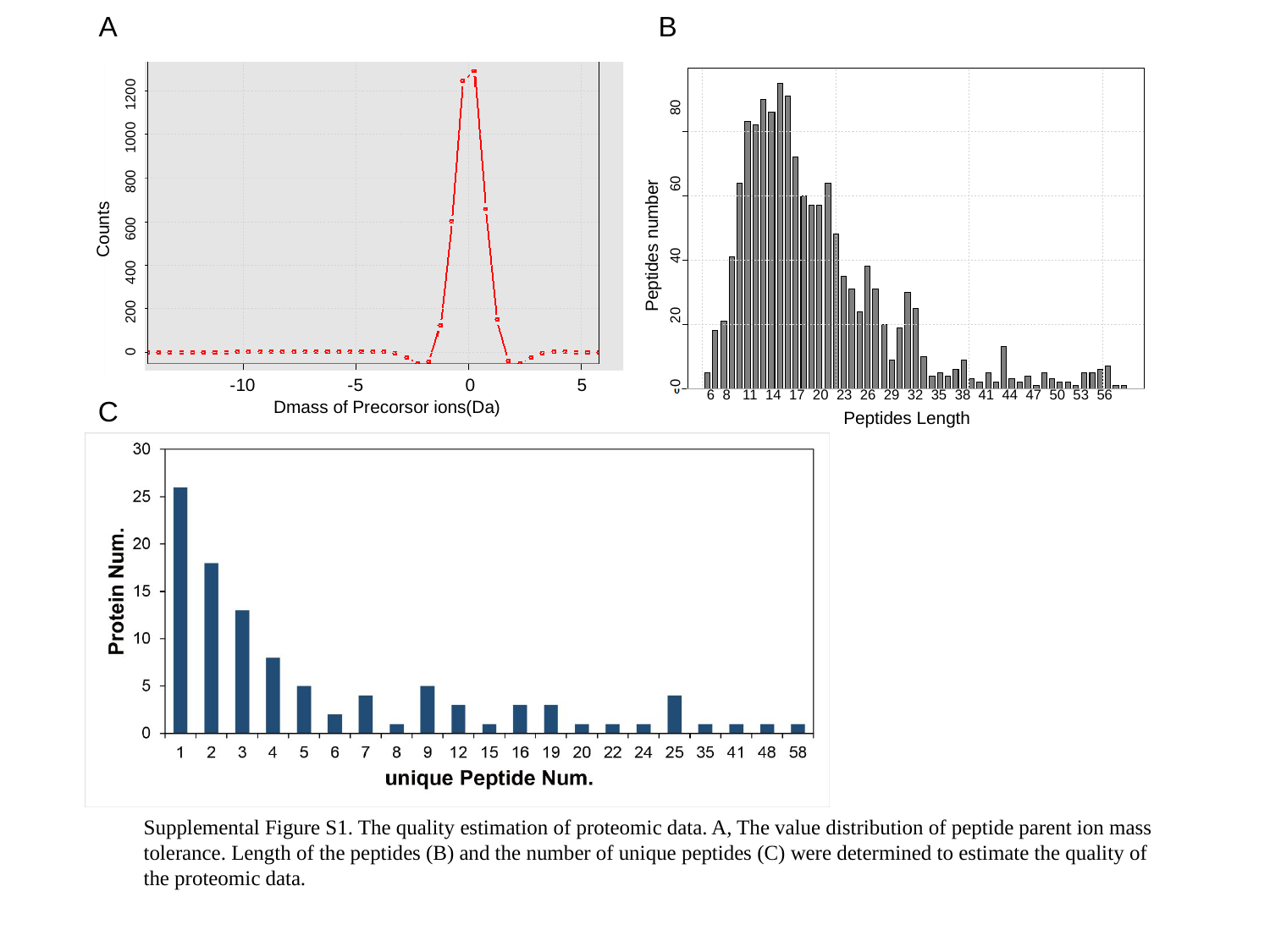

A
B
0 200 400 600 800 1000 1200
Counts
0 20 40 60 80
Peptides number
-10 -5 0 5
6 8 11 14 17 20 23 26 29 32 35 38 41 44 47 50 53 56
Dmass of Precorsor ions(Da)
Peptides Length
C
Supplemental Figure S1. The quality estimation of proteomic data. A, The value distribution of peptide parent ion mass tolerance. Length of the peptides (B) and the number of unique peptides (C) were determined to estimate the quality of the proteomic data.
