# Supplementary material for: Analysis of the Composition of Deinagkistrodon acutus Snake Venom Based on Proteomics, and Its Antithrombotic Activity and Toxicity Studies
Source: Molecules. 2022 Mar 29;27(7):2229. doi: 10.3390/molecules27072229 (PMC9000436; doi:10.3390/molecules27072229)
Supplement: Supplementary file 1 [file molecules-27-02229-s001.zip › molecules-1609315-supplementary/Supplemental FileS1 snak_Map.html]

snak


| Pathway | Diff Proteins with pathway annotation (38) | Pathway ID |
| --- | --- | --- |

| Pathway | Proteins |
| --- | --- |
